# Pre-exposure prophylaxis during the SARS-CoV-2 pandemic: can PrEP prevent COVID-19-related symptoms?

**DOI:** 10.1017/S0950268820002253

**Published:** 2020-09-28

**Authors:** Danilo Euclides Fernandes, Paulo Roberto Abrão Ferreira, Gianna Mastroianni Kirsztajn

**Affiliations:** 1 Department of Medicine (Nephrology), Universidade Federal de São Paulo (UNIFESP), Sao Paulo, Brazil; 2 Department of Medicine (Infectology), UNIFESP, Sao Paulo, Brazil

**Keywords:** 2019 novel coronavirus disease, flu-like, Influenza, Human, Pandemics, Pre-Exposure Prophylaxis (PrEP), Coronavirus, SARS-CoV-2, COVID-19

## Abstract

It has been speculated that some drugs can be used against SARS-CoV-2. As for antiretrovirals, the follow-up of pre-exposure prophylaxis (PrEP) users during the coronavirus disease 2019 (COVID-19) outbreak may help to understand the potential protective effect of PrEP against SARS-CoV-2. We aimed to identify associations between oral PrEP use and COVID-19-related symptoms self-reporting. Phone call interviews or digital investigation (through WhatsApp^®^ or e-mail) about oral PrEP regular use, social distancing, exposure to suspected or confirmed cases of SARS-CoV-2 infection and COVID-19-related symptoms. Among 108 individuals, the majority were cisgender, white and gay men. Although most of the individuals engaged in social distancing (68.52%), they kept on taking PrEP (75.93%). Few people have had contact with suspected or confirmed cases of COVID-19 (12.04%), but some had COVID-19-related symptoms the month before the interview (27.78%) including rhinorrheoa (56.67%), cough (53.33%), asthaenia (50.00%) and headache (43.33%). Also, oral PrEP was associated with lower self-reporting COVID-19-symptoms (OR 0.26, 95% CI 0.07–0.96, *P* = 0.04; *h* = 0.92) even after controlling confounders as social distancing, age, body-mass index and morbidities . In our sample, the regular use of oral PrEP was associated with lower self-reporting of COVID-19-related symptoms during the outbreak in São Paulo, Brazil.

## Introduction

In March 2020, the World Health Organization (WHO) declared a pandemic situation of SARS-CoV-2 after several cases of pneumonia worldwide [1]. The milder and most frequent symptoms were identified as flu-like ones such as cough, fever, rhinorrhoea and headache [2]. Anosmia and ageusia were also included in this list of COVID-19-related symptoms [3].

In Brazil, by the end of February, close to carnival celebrations, the first suspected cases of COVID-19 started to appear [4] and were further confirmed by the reverse transcription-polymerase chain reaction (RT-PCR) test. Despite the country's president statements, governors of Brazilian states and the previous Ministry of Health, Dr. Luiz Henrique Mandetta, instituted social distancing, following the worldwide campaign proposed by the WHO to flatten the curve of COVID-19 incidence, also avoiding the health system collapse. Similar to other cities around the world, São Paulo – the most populous city in Brazil – changed its routine to obey worldwide ‘stay home’ recommendations, preventing the virus from spreading.

Oral pre-exposure prophylaxis (PrEP) has been available outside of clinical trials in Brazil since 2018 as a combined HIV prevention strategy, primarily for men who have sex with men, transgender women, sex workers and serodiscordant couples [5, 6]. Oral PrEP tablets contain 300 mg tenofovir disoproxil fumarate (TDF) and 200 mg emtricitabine (FTC). Users are required to come back to their prescribing clinic every 3 to 4 months for clinical and laboratory follow-up.

This study aimed to identify associations between oral PrEP (TDF/FTC) use and COVID-19 symptoms self-reporting. These associations are intended to comprise preliminary data to support further investigation of the potential protective effect of PrEP against COVID-19 symptoms.

## Materials and methods

This is a case-control and unique centre study developed at Universidade Federal de São Paulo (UNIFESP), Brazil. This protocol was approved by the Ethical Committee on Research of the UNIFESP (CAAE 96087918.9.0000.5505) and was developed according to the principles expressed in the Declaration of Helsinki. All participants provided informed consent through digital agreement via online survey or WhatsApp^®^.

We enrolled PrEP users from our on-going cohort [7]. Inclusion criteria were regular use of oral PrEP for 6 months, at minimum, or discontinuation due to social distancing only (from March 2020 on, when social distancing was recommended in Brazil). All individuals who did not use PrEP regularly for other reasons were excluded.

### Procedures

**Fig. 1.**
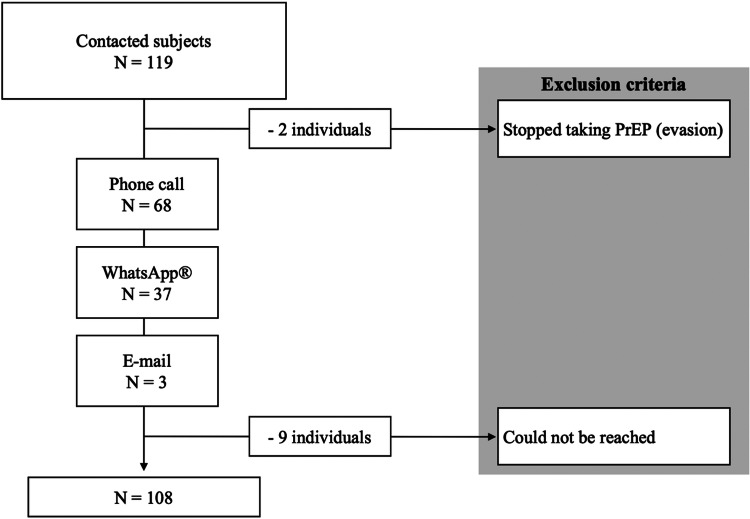
Enrollment flowchart.

We obtained demographic and clinical data from the individual's chart at their prescribing clinic. Then, we contacted the individuals from 1st to 3rd April 2020 through phone call, WhatsApp^®^ or e-mail as shown in Figure 1, and invited them to respond to a few questions about oral PrEP use during the COVID-19 pandemic in Brazil. The questions were:

- Have you been taking PrEP regularly?
- Have you been engaged in social distancing for the past 2 weeks?
- Have you had contact with suspected or confirmed cases of COVID-19?
- Have you had COVID-19-related symptoms over the last month? If yes, which ones (rhinorrhoea, cough, asthaenia, headache, sore throat, fever, decreased taste, dyspnoea, loss of smell, diarrhoea)?

We considered each of the COVID-19-related symptoms separately and then aggregated them together to form a binary measure (e.g. experienced symptoms/did not experience symptoms). The first three questions were also taken as a dichotomous variable (yes or no).

### Statistical analysis

Data were analysed by a biostatistician using SPSS Statistics^®^, version 26. We set self-report of COVID-19-related symptoms as our primary outcome and used it as the dependent variable in logistic regression, which was controlled for confounders in four models: (1) social distancing; (2) social distancing and age; (3) social distancing, age and body mass index and (4) social distancing, age, body-mass index and comorbidities. As body-mass index [8] and comorbidities [9–11] are described to be risk factors for COVID-19, we decided to include them in our models. We did not include education as an independent variable because of the characteristics of our sample, in which the majority is highly educated. We used the PROCESS 3.5 SPSS plug-in to run the pathway analyses [12]. As we did not calculate sample size previously, we used Cohen's methods to estimate the effect size. We set *α* = 0.05 and confidence interval = 95% for all the tests. Some sample characteristics and COVID-19-related symptoms that are shown in descriptive statistics were not used in the regression analyses.

## Results

We excluded nine individuals who could not be reached and two individuals who stopped taking oral PrEP before the COVID-19 pandemic. Our final sample accounted for 108 subjects.

The majority of the individuals were cisgender (95.37%), white (86.11%), had a normal body-mass index (62.04%), and were gay men (90.74%). They were, on average, 33.9 (±8.36) years old and highly educated (96.30%). They had on average 44.5 (±72.83) sexual partners over the 6 months before the interview. Most of them had no morbidities (71.30%) and have not been taking other medications (56.48%). Few of them were sex workers (11.11%). Also, few of them (6.48%) were highly exposed to risky situations for SARS-CoV-2 infection due to their occupation as physicians in the frontline against COVID-19 (Table 1).

**Table 1.** Socio-demographic characteristics of the PrEP users

Gender, *n* (%)		
Cisman	103	(95.37)
Ciswoman	4	(3.70)
Transwoman	1	(0.93)
Ethnicity, *n* (%)		
White	93	(86.11)
Mixed	13	(12.04)
Black	2	(1.85)
Age, mean (s.d.), years	33.9	(8.36)
Sexual orientation, *n* (%)		
Homosexual	98	(90.74)
Bisexual	5	(4.63)
Heterosexual	5	(4.63)
Education, *n* (%), years		
<12	4	(3.70)
≥12	104	(96.30)
Weight, mean (s.d.), kg	75.9	(11.58)
Height, mean (s.d.), cm	175.9	(6.88)
BMI category, *n* (%), kg/m^2^		
<18.5	0	(0.00)
18.5–24.9	67	(62.04)
25–29.9	31	(28.70)
≥30	9	(8.33)
Drug or tobacco use, *n* (%)		
Alcohol	93	(86.11)
Marijuana	40	(37.04)
Tobacco	37	(34.26)
Sex workers, *n* (%)	12	(11.11)
Sexual partners in the last 6 months, mean (s.d.)	44.5	(72.83)
Other diseases, *n* (%)	31	(28.70)
Other medications, *n* (%)	47	(43.52)

*n*, sample; BMI, body mass index; s.d., standard deviation; COVID-19, coronavirus disease 2019; PrEP, pre-exposure prophylaxis.

Although most of the individuals engaged in social distancing (68.52%), they kept on taking oral PrEP (75.93%). Fewer people had had contact with suspected or confirmed cases of COVID-19 (12.04%) and some individuals reported COVID-19-related symptoms the month before the interview (27.78%) – rhinorrhoea (56.67%), cough (53.33%), asthaenia (50.00%) and headache (43.33%) – and one of the subjects took oseltamivir without a medical prescription (Table 2).

**Table 2.** Answers to the survey and the most frequent COVID-19 (flu-like) symptoms

Questions	*n*	(%)
Have you been taking PrEP regularly?	82	(75.93)
Have you been in social distancing for the past 2 weeks?	74	(68.52)
Have you had contact with someone suspect of confirmed COVID-19?	13	(12.04)
Have you had COVID-19-related symptoms over the last month?^a^	30	(27.78)
Rhinorrhoea	17	(56.67)
Cough	16	(53.33)
Asthaenia	15	(50.00)
Headache	13	(43.33)
Sore throat	11	(36.67)
Fever	8	(26.67)
Ageusia	4	(13.33)
Dyspnoea	4	(13.33)
Anosmia	4	(13.33)
Diarrhoea	3	(10.00)

*n*, sample; COVID-19, coronavirus disease 2019; PrEP, pre-exposure prophylaxis.

a And/or additional symptoms described in COVID-19 infection.

Cohen's *h* (*h* = 0.92) supports the power of our sample towards the primary outcome: self-reporting of COVID-19-related symptoms. Logistic regression test showed statistical significance for PrEP regular use (odds ratio (OR) 0.26, 95% confidence interval (CI) 0.07–0.96, *P* = 0.04; model 0) even after controlling for confounders – social distancing, age, body-mass index and comorbidities – as shown in Table 3. Multiple logistic regressions showed that social distancing did not interfere with oral PrEP on self-reporting COVID-19-related symptoms. Additionally, a pathway analysis confirmed no moderation effect between oral PrEP regular use and the presence of the symptoms.

**Table 3.** Simple and multiple logistic regressions (outcome variable: self-reporting of COVID-19-related symptoms)

	Model 0			Model 1			Model 2			Model 3			Model 4		
	*P*	OR	(95% CI)	*P*	OR	(95% CI)	*P*	OR	(95% CI)	*P*	OR	(95% CI)	*P*	OR	(95% CI)
Oral PrEP use	0.04	0.26	(0.07–0.96)	0.04	0.25	(0.06–0.99)	0.04	0.26	(0.06–0.99)	0.04	0.24	(0.06–0.98)	0.04	0.24	(0.06–0.95)
Social distancing	–	–	–	0.81	1.13	(0.40–3.16)	0.80	1.14	(0.40–3.20)	0.83	1.11	(0.39–3.13)	0.85	1.10	(0.39–3.11)
Age	–	–	–	–	–	–	0.83	0.99	(0.94–1.04)	0.86	0.99	(0.94–1.05)	0.89	0.99	(0.09–1.05)
Body-mass index	–	–	–	–	–	–	–	–	–	0.86	0.98	(0.85–0.11)	0.98	0.99	(0.86–1.15)
Morbidities	–	–	–	–	–	–	–	–	–	–	–	–	0.58	1.32	(0.48–3.63)

*P*, *P*-value; OR, odds ratio; CI, confidence interval.

Model 1: adjusted for social distancing.

Model 2: adjusted for social distancing and age.

Model 3: adjusted for social distancing, age and body-mass index.

Model 4: adjusted for social distancing, age, body-mass index and comorbidities.

## Discussion

Our study presents preliminary data that intended to support further investigation on the potential protective effect of oral PrEP against COVID-19 symptoms.

Oral PrEP has been a combined HIV prevention strategy [5, 13] for both individual and populational HIV prophylaxis [14]. As we observed in this study, PrEP users tend to be young adults, highly educated and healthy [5, 13, 15] and their main goal is to stay free of diseases [16]. Our findings suggest that the regular use of PrEP is associated with lower self-reporting of COVID-19-related symptoms. We recognise that many aspects should be taken into account, but we found no evidence that other diseases [9–11] and body-mass index [8] affected the frequency of COVID-19-related symptoms in our sample.

Despite promising *in vitro* results on antiretroviral drug effects against SARS-CoV-2 infection [17, 18], clinical research does not confirm their potential *in vivo* [19]. Some of those drugs can inhibit the viral RNA polymerase, which is vital for the SARS-CoV-2 cycle [17], but none of them changed significantly the course of COVID-19 or its mortality rate [19]. Preliminary findings as ours help to guide epidemiological, experimental and clinical investigations for such a condition with no golden-standard treatment as COVID-19.

Few people stopped taking oral PrEP during social distancing, which supports that PrEP users are highly adherent to the treatment [15], even on reduced sexual exposure.

Self-reported symptoms included rhinorrhoea, cough, asthaenia, headache, fever, ageusia and/or anosmia, which have been associated with SARS-CoV-2 infection [2, 10]. Low prevalence of dyspnoea and the absence of hospitalisation suggest mild symptomatology.

Daily exposure to SARS-CoV-2 at the COVID-19 frontline put health care workers at higher risk of infection [20]. The seven physicians we have on our sample kept on taking oral PrEP and reported only mild symptoms as rhinorrhoea, cough, anosmia and/or ageusia, regardless of their daily risky routine.

Our analyses showed that self-reported COVID-19-related symptoms were independently associated with oral PrEP regular use, even after controlling for confounders such as age [21, 22], body-mass index [11], and comorbidities [21, 22]. We confirmed that social distancing did not moderate PrEP protective effect through the pathway analysis and all the logistic regression models confirmed that oral PrEP reduced self-reporting of COVID-19 symptoms in our sample.

## Limitations

Our findings suggest that oral PrEP associates with less self-reports of COVID-19-related symptoms, but we cannot state that oral PrEP reduces the risk of SARS-CoV-2 infection because a case-control study is inappropriate to do so. Additionally, we could not confirm SARS-CoV-2 infection by RT-PCR or serology due to non-availability at the onset of the outbreak in Brazil, so we had to rely on the clinical presentation of COVID-19. Regardless of the limited sample size, we had powerful Cohen's coefficients and our multiple regression analysis remained strong enough to accept the independent variables we included.

## Conclusion

In our sample, the regular use of oral PrEP was associated with lower self-reporting of COVID-19-related symptoms during the outbreak in São Paulo, Brazil.

## Data Availability

The datasets generated during and/or analysed during the current study are available from the authors on reasonable request.
